# Factors associated with dropout from treatment for eating disorders: a comprehensive literature review

**DOI:** 10.1186/1471-244X-9-67

**Published:** 2009-10-09

**Authors:** Secondo Fassino, Andrea Pierò, Elena Tomba, Giovanni Abbate-Daga

**Affiliations:** 1 Eating Disorders Centre, Department of Neuroscience, University of Turin, Via Cherasco 11, 10126 Turin, Italy; 2 Mental Health Department ASL TO 4, Mental Health Centre, Via Blatta 10, Chivasso, 10034 Turin, Italy; 3 Department of Psychology, University of Bologna, 40127 Bologna, Italy

## Abstract

**Background:**

Dropout (DO) is common in the treatment of eating disorders (EDs), but the reasons for this phenomenon remain unclear. This study is an extensive review of the literature regarding DO predictors in EDs.

**Methods:**

All papers in PubMed, PsycINFO and Cochrane Library (1980-2009) were considered. Methodological issues and detailed results were analysed for each paper. After selection according to inclusion criteria, 26 studies were reviewed.

**Results:**

The dropout rates ranged from 20.2% to 51% (inpatient) and from 29% to 73% (outpatient). Predictors of dropout were inconsistent due to methodological flaws and limited sample sizes. There is no evidence that baseline ED clinical severity, psychiatric comorbidity or treatment issues affect dropout. The most consistent predictor is the binge-purging subtype of anorexia nervosa. Good evidence exists that two psychological traits (high maturity fear and impulsivity) and two personality dimensions (low self-directedness, low cooperativeness) are related to dropout.

**Conclusion:**

Implications for clinical practice and areas for further research are discussed. Particularly, these results highlight the need for a shared definition of dropout in the treatment of eating disorders for both inpatient and outpatient settings. Moreover, the assessment of personality dimensions (impulse control, self-efficacy, maturity fear and others) as liability factors for dropout seems an important issue for creating specific strategies to reduce the dropout phenomenon in eating disorders.

## Background

Eating disorders (EDs) are serious and complex mental diseases, and their pathogenesis includes individual psychobiological vulnerability (genetic) and shared (culture) or unshared (life events) environmental factors [1-3].

Treatment of EDs is complex and multidisciplinary [1], and the rate of dropout is very high: up to 70% of ED patients drop out of outpatient treatment [4], whereas reported dropout rates for patients with anorexia nervosa (AN) from specialised inpatient eating disorder programmes range from 20.2% to 51% [5].

The term "dropout" has been used to describe both the unilateral ending of regular treatment by a patient and the decision for administrative discharge made by a treatment team. Clinicians and researchers working with EDs have long recognised the problem of treatment dropout and its implications for long-term recovery [6,7], but for many years, the dropout phenomenon in EDs was usually reported only as quantitative data within the context of clinical trials. Only recently has the relevance of this phenomenon been fully understood [8].

We located reviews of limited interest about this phenomenon [9-11]. The review by Mitchell [9] included only selected trials intended to study the efficacy of treatments. It is likely that the researchers made several efforts to reduce the dropout phenomenon in these trials and tried to include only highly motivated subjects. Therefore, it is likely that the dropout rate would have been higher in naturalistic studies. Moreover, predictors of dropout were seldom analysed in these studies. Mahon's review [10] focused only on factors leading patients with AN to drop out, whereas studies of bulimia nervosa (BN) and eating disorders not otherwise specified (ED-NOS) were neglected. Bacaltchuk and Hay [11] analysed the dropout (attrition) rate of bulimic patients from treatment with SSRIs.

A recent paper by Wallier and colleagues [5] is focused on dropout from inpatient treatment. The authors revealed seven studies of interest [6,12-17], and critically discussed the methodological flaws and the inconsistency of the results. Although this review is interesting, it is only a partial analysis of the literature. Most individuals with EDs are treated in an outpatient setting, and hospitalisation becomes necessary when outpatient programs fail [18]. In fact, the inpatient population shows more severe overall eating and psychiatric psychopathology. A comprehensive review of the dropout phenomenon requires the inclusion of the outpatient setting.

The study of the features of patients with EDs who do not complete their treatment program seems to be crucial for many reasons: (a) the high incidence of this phenomenon largely decreases the power and generalisability of the results of clinical trials [8]; (b) most of the non-completer inpatients affected by EDs have poorer prognoses [15]; (c) most of the non-completers tend to be referred to specialised centres again after months or years, when their psychopathology is more severe and the course of their illness tends to be chronic [19]; (d) some studies suggest that premature dropout is a risk factor for relapse within the first year after hospitalisation [7]; (e) although the estimates are somewhat inaccurate, dropout appears to be a very expensive phenomenon in terms of the direct and indirect costs of chronic EDs [20]; and (f) prediction of dropout is an important step toward developing interventions to reduce it and to improve the treatment of engaged and completer subjects [14,21-23].

Therefore, the aim of this review is to summarise the body of evidence in this research area, to emphasise its clinical implications and to highlight some critical points.

## Method

### Data sources and study selection

The following inclusion criteria were used to select studies assessing the factors related to early interruption of clinical interventions in subjects with EDs: (a) inclusion in at least one of three databases, MEDLINE, PsycINFO and Cochrane Library, from January 1980 to January 2009; (b) studies with the specific aim of analysing the dropout phenomenon (the trials describing only the number of and motivations for dropouts were not considered adequate for inclusion in this review); (c) original articles published as full papers or brief reports (no letters); (d) inclusion of at least one form of psychological treatment in addition to nutritional support and/or medication for inpatient treatment; (e) studies including adult or mixed adolescent/adult samples - the reasons why younger patients adhere to treatment could be different from those of older, adult patients because parental approval is legally required for treatment or interruption of treatment of minor patients; and (f) studies published in the English language.

The following medical subject headings or key words were used: dropout, termination, adherence, attrition, eating disorders (EDs), bulimia nervosa (BN), anorexia nervosa (AN). The Cochrane Library and PsycINFO database were searched with the same key words.

A computerised search and a manual search from the references sections of included papers were performed.

The diversity of sample compositions, treatments, definitions of dropouts, settings of treatment and predictors analysed made it impossible to conduct a meta-analysis of all of the available studies.

Therefore, this is a descriptive, comprehensive and critical review of the features of the studies on this topic.

### Data extraction

An ad hoc form was designed for data extraction, including: (a) authors; (b) therapeutic setting (inpatient or outpatient) and ED diagnosis; (c) number of subjects included and dropout rate; (d) two main features of the samples (age, illness duration); (e) type of treatment; (f) type of dropout; and (g) predictors of dropout.

All of the studies reviewed are reported in Additional file 1 and Additional file 2.

### Dropout definition

The studies reviewed define dropout in different ways. The terms "dropout", "attrition", and "premature termination" are often used interchangeably to describe this phenomenon.

Two features appear to characterise the dropout definition: (a) patient-initiated or staff-initiated discharge or interruption; and (b) percentage of the treatment program completed (timing).

Some studies define dropout (type A; DO-A) as a non-consensual interruption of treatment on the basis of the patient's decision; several authors define "completers" as those patients who interrupt treatment after completing 75% of the program, whereas others consider "dropout" to be the interruption of treatment at any time during the program. This type of dropout (DO-A) is often operationally defined as "having attended at least one session for diagnostic assessment or treatment and discontinuing the assessment or treatment process on the patient's own initiative by failing to attend any further planned visit"[24].

Other studies (inpatient setting) define dropout in a different way (type B; DO-B): the patients are discharged if they do not reach the purposed aims at intake (BMI > 19 or interruption of purging behaviours) because not reaching the expected goals is considered by the authors as an opposition to treatment comparable to dropout [5]. In this case, the interruption is a staff-initiated dropout.

We included in this review both of these types of dropout (DO-A; DO-B). Moreover, some researchers (Additional files 1 and 2) distinguish early dropouts (E-DO) and late dropouts (L-DO). "Early dropout" refers to an interruption of treatment after 2-3 sessions or within the first month; "late dropout" refers to the interruption of treatment after more than one month.

Other authors (inpatient setting) consider "early dropouts" to be subjects discharged at or below 80% of IBW (Ideal Body Weight) and "late dropouts" those discharged at or above 81% of IBW [5,15].

The inability to initiate the treatment and the refusal of treatment suggested at intake are defined as "failure to engage" (FE), but only two studies considered this phenomenon [25,26].

### Papers of some interest excluded from the review

One paper was excluded because the authors did not report a clear definition of dropout [27]. These researchers showed that the dropouts from an outpatient cognitive behavioural treatment programme were characterised by more severe bulimic cognitions and greater impulsivity, but it was not possible to identify clinically useful predictors.

Four additional papers were excluded because they included only adolescent subjects younger than 18 years [16,23,28,29].

One paper was excluded because it is not a study of the predictors of dropout [30], but an analysis of what happened to ED subjects who had dropped out of therapy 2-5 years earlier. The investigators found an unexpected result: 71% of these subjects were "improved", and no deaths were recorded. Particularly, subjects with shorter illness durations and without follow-up treatment were more likely to be in this improved group.

One paper was excluded because of limitations in the sample size (only eight subjects) and because only qualitative analysis was carried out [31].

Finally, four papers were excluded because of considerable methodological limitations [32-34] concerning data analysis, sample selection or study design.

## Results

### Search results

The full text of 52 articles retrieved from PubMed, PsycINFO and Cochrane Library searches was screened. Only 37 papers out of these 52 were considered pertinent, and 26 of these were retained on the basis of the inclusion criteria (eleven were excluded, as described in the preceding section). The main characteristics of the 26 studies are listed in Additional files 1 and 2.

Only three papers studying the dropout phenomenon used a randomised controlled trial (RCT) methodology [8,35,36], while the others were retrospective and non-controlled studies.

The dropout rates were 20.2-51% for inpatient treatment (Additional file 1) and 29-73% for outpatient treatment (Additional file 2).

### Sample composition

The numbers of subjects included in the studies varied widely (Additional files 1 and 2), ranging from 20 to 261 (mean 110.2; SD = 57.4). Some studies also included males, but they always represented a negligible portion of the sample (0-2%), making a comparison based on gender impossible. The size of the sample and the diagnosis/gender composition depend on the characteristics of the setting (inpatient, outpatient) and on the duration of observation, whereas the power of the study is not considered in the majority of these studies.

Differentiating by setting, the mean number of subjects included in inpatient studies was 143.5 (SD = 44.6), with a range between 77 [17] and 213 [12], and in outpatient studies the mean was 92.5 (SD = 56.5), with a range between 20 [22] and 261 [37].

The mean age of patients included was 24.7 years (SD = 7.4 years) for the nine inpatient studies and 23.9 years (SD = 6.1 years) for the fifteen outpatient studies (in two studies, this datum was not mentioned). The mean illness duration was 6.4 years (SD = 6.1 years) for the nine inpatient studies and 5.5 years (SD = 3.5 years) for the seven outpatient studies that mentioned this datum. Of course, age and illness duration are higher in subjects included in inpatient studies because subjects who need inpatient treatment are more severely affected by the disorder and often are more resistant to treatment. Moreover, some of these studies included mixed samples of patients at first admission and those who needed more than one hospitalisation during the inclusion period [5].

### Diagnosis

With regard to DSM-IV or DSM-III (-R) ED diagnoses, six studies (28.7%) included only subjects with anorexia nervosa (AN), eight (38%) only subjects with bulimia nervosa (BN), and seven (33.3%) both AN and BN subjects (or ED-Not Otherwise Specified, ED-NOS). In particular, only five studies included subjects with a diagnosis of ED-NOS (two inpatient and three outpatient).

### Definition and timing of dropout

In terms of the definitions of dropout described above, 19 of the reviewed studies evaluated "type A" dropout (73%), two "type B" dropout (8%), three both types A and B (11%), two failure to engage (8%; one only the failure to engage and one DO-A + FE), and only two discriminated between "early" and "late" dropout (8%).

### Therapeutic setting

#### Inpatient setting

Nine studies (34.6%) analysed the dropout phenomenon in an inpatient setting [6,12-15,17,18,37,38]. In one study, subjects applied to an outpatient setting after discharge from inpatient treatment [35].

Given that female patients with AN need inpatient treatment with greater frequency than subjects with BN, only two of the studies mentioned above included women with BN [18,38].

The treatment programs described in these nine studies are largely equivalent: they all share a nutritional and psychological treatment approach. Medication was also used in many patients. Most of these studies included family therapy or counselling for the parents of patients in the treatment program.

#### Outpatient setting

Two studies out of the 17 dealing with an outpatient setting included only subjects with AN [8,39], whereas the others included patients with BN (n = 8) or mixed samples of subjects with EDs (n = 7). The types of treatment are highly variable, but, as per the inclusion criteria, all of these studies involved at least one kind of psychological treatment (individual or group, cognitive-behavioural or psychodynamic) in addition to nutritional or medication treatments. A small subset of these studies had a structured nutritional approach (7/17).

### Predictors

The studies reviewed took very different approaches to studying the predictors of dropout in the treatment of subjects with EDs. Some studies aimed to research personal, clinical and psychological predictors of dropout with tailored tools, whereas others did not use a standardised and validated assessment, but only retrospective chart review and clinical records.

Additional files 1 and 2 show all of the predictors found in the studies included in this review as well as all of the questionnaires used to research the predictors of dropout. Almost all of the studies used ED-related inventories. Only two studies accurately investigated Axis I and II comorbidity with the SCID-I and II [8,13].

Several of the studied predictors were found to affect dropout rate, but not all of the variables were studied by all of the authors. Therefore, a small number of baseline characteristics were found to predict dropout in two or more independent studies. In the following, we list all of the variables that were found in at least one study to be related to a higher risk of dropout:

#### ED Diagnosis

The binge-purging subtype of AN was found to predict dropout in four independent inpatient studies (4/6), whereas BN predicted dropout in two outpatient studies (2/7). Three inpatient studies and ten outpatient studies presented only a single diagnostic group.

#### Psychological traits and personality

Higher harm avoidance (1/3), lower persistence (1/3) of temperament, lower self-directedness (2/3) and cooperativeness (2/3) of character; low self-esteem (2/9); higher interpersonal distrust, difficulties relating to others (2/3), maturity fear (4/15), borderline personality disorder (2/2), and broadly defined borderline traits (5/15); patient's higher expectations about treatment (2/2).

#### Eating symptoms or attitudes

Higher drive for thinness (2/22), higher bulimic attitudes (2/22), higher body dissatisfaction (2/22), higher perfectionism (1/22), longer illness duration (1/22).

#### Nutritional status and eating disorder history

Higher body mass index (BMI) at intake (1/19), lower BMI at intake (1/19), later age of onset (1/19).

#### Demographic characteristics

Lower age at intake (1/22), older age at intake (1/22), lower educational level (1/16), employment status (2/10).

#### Psychopathological status

Higher depression (1/14), lower depression (1/14), higher hostility (1/5), impulsivity (2/11) and dissociation (1/1); poorer anger management (2/2), higher number of previous psychiatric treatments (2/6).

#### Life events

Higher rate of early life events, such as sexual abuse (3/3) or other life events (1/2).

#### Family environment

Higher expressed emotion (1/4) and level of psychopathology in parents (1/2).

#### Type of treatment

Only five studies compared more than one treatment and included this variable in the analysis of the possible predictors of dropout. In one study, family therapy was more likely to lead to dropout in BN subjects [35].

## Discussion

This review of the literature concerning the dropout phenomenon in eating disorders revealed two major findings: 1) the problem of dropout in treatment of EDs is confirmed as a major topic since it occurs in 20-51% of inpatients and 29-73% of outpatients; 2) the number of predictors found as significant in more than two studies is very small (AN binge eating/purging type, maturity fear, broadly defined borderline traits and early life events). Some interesting results emerged concerning the assessment of these traits within a psychobiological model of personality (TCI).

Rates of dropout may be influenced by numerous methodological issues. In fact, the available literature is biased by small samples with different compositions, poor statistical power and a substantial lack of agreement about the definition of dropout. Particular difficulties emerged in the interpretation of dropout in inpatient studies because the administrative staff initiated discharge and termination decisions by the patient were not always explicitly mentioned. Moreover, the criteria for administrative discharge vary between the inpatient studies in relation to clinical practice [5].

Regarding the lack of consistent predictors of dropout, the small sample sizes in relation to the large number of variables investigated and the tendency to study only the variables identified in previous studies or only new and specific psychological and personality dimensions are the major factors making a quantitative analysis difficult. For example, only the severity of the eating disorder and socio-demographic features were studied by all of the researchers. Both of these types of predictors showed inconsistent results both in inpatient and outpatient studies.

The factor most frequently examined and identified as a predictor of dropout was the AN binge-purging subtype in inpatient studies [5]. AN-BP subjects may be more impulsive and more unstable [40]. It is thus possible that rigid inpatient treatments requiring early behavioural changes are not well suited for these patients without a previous motivational intervention [41,42]. Moreover, the AN-BP subtype is a well-defined predictor of worse outcome for anorexia nervosa [43], and these patients have higher mortality rates.

Of the seven outpatient studies that considered more than one diagnostic group (AN, BN, ED-NOS), bulimic subjects were identified as more likely to drop out in two independent studies [4,35]. Therefore, as this predictor is somewhat consistent with the previous one (anorexia binge-purging subtype/AN-BP in inpatient studies), it seems that subjects with impulsive behaviours have a higher risk of not completing their treatment.

Two related psychological traits emerged as predictors of dropout in at least two studies (outpatient): 1) Maturity fear, which expresses the fear of abandoning the confidence of preadolescence and facing the responsibilities of adulthood [44], and 2) impulsivity.

It is important to underscore that higher levels of impulsivity and maturity fear are typical of patients with low self-esteem and "borderline" features [44]. Borderline personality disorder or "borderline traits" have been identified as predictors of dropout in EDs in at least seven studies included in this review [12-14,25,26,39,45] and also in personality disorder treatment [46]. These subjects have difficulties with the regulation of self-esteem and affect in addition to problems with separating and with impulse control [47].

Personality functioning was considered in different ways by 46% (12/26) of the authors, and most of these authors showed that some personality traits, dimensions or patterns of functioning play an important role in dropout.

Numerous previous studies have demonstrated with the Temperament and Character Inventory (TCI) [48] that harm avoidance, novelty seeking and self-directedness may have a pathogenetic role in EDs [49]. Moreover, a high level of harm avoidance (HA) seems to be an endophenotype related to vulnerability to the development of EDs [50]. The self-directedness (SD) scale of the TCI, which describes the ability of the individual to define and pursue goals and to have mature and balanced relationships [48], is related to the severity of BN [51], to anger management in ED [40], and to the response to psychological treatment [52].

Only three studies in outpatients and in inpatients [18,39,45] investigated the personality dimensions of temperament and character (assessed with the TCI- Temperament and Character Inventory) as predictors of dropout: Fassino and coworkers identified a dimension of temperament (higher harm avoidance in AN) and two of character (lower self-directedness and cooperativeness in AN and BN) as predictors of dropout in outpatients with EDs, whereas Dalle Grave and colleagues [18] found a relation between dropout and a lower persistence of temperament in inpatients.

Axis II personality disorders (PD) received little attention. Researchers failed to find any consistent association between PD and dropout, in contrast with the evidence that the presence of Axis II comorbidity leads to difficulties in the psychotherapeutic relationship [41] in EDs.

The results regarding mood state are controversial in both inpatient and outpatient studies. Overall, general psychopathology seems to play a negligible role in the early interruption of treatment in EDs, whereas two studies showed that the number of previous hospitalisations and psychiatric treatments predicted dropout [13,53]. These two variables can decrease the motivation to finish treatments, particularly in residential programs.

Another variable identified in two studies was difficulties with anger management [39,45]. Recently, investigators have paid greater attention to anger and aggressiveness management in EDs [54]. Anger management seems to play a pathogenetic role in both AN and BN [40]. Similar data illustrate the role of hostility in dropping out [55]. Further studies in this field are required.

As to the type of treatment, only one study showed that family therapy is more likely to lead to an early interruption of treatment in BN subjects [35]. Other studies [6,8,24] did not support this result. Further investigations in this area are needed because the settings and programs of treatment are very different in the studies reviewed both for inpatient [5] and outpatient treatment: eclectic vs. non-eclectic, integrated vs. not integrated, psychological vs. combined treatment (nutritional and psychological), combined vs. sequential treatment [6].

This topic is of extreme interest because it is conceivable that administration of treatments in sequential order [56] may allow clinicians to address issues that may hinder adequate collaboration with treatment. This as yet untested approach may also lead to individualised strategies based on the fact that a treatment that is helpful to many patients may not be suitable or may even be harmful in a specific subgroup [57].

The inadequacies of standard clinical assessment in psychiatry have been recently outlined [58]. The staging method, whereby a disorder is characterised according to its seriousness, extension and features, has achieved widespread currency in medicine, but it is currently neglected in psychiatry [58,59]. Staging has the potential for improving the logic and timing of interventions, just as it does in many complex and serious medical disorders. Prochaska [60] developed a staging system for readiness to change (precontemplation, contemplation, preparation, action, maintenance, termination) that may find applicability in EDs [61]. The sequential model may again be appropriate for improving readiness to change before treatment of EDs, thus potentially yielding a lower rate of dropout. There have been only a few attempts to evaluate this treatment strategy, and data about the role of stage of change in the dropout phenomenon are still preliminary [62]. Moreover, a poor motivation for and/or dissatisfaction with the treatment are usually found in patients with psychiatric disorders who drop out of cognitive behavioural therapy [63].

It is also important to understand whether dropout is always a negative event for patients and their families, and follow-up studies in this area are needed. Resistance to treatment does not necessarily mean resistance to change [6]. Moreover, it is useful to remember that "showing up for treatment does not necessarily mean compliance with treatment or following treatment recommendations" [8]. In fact, preliminary results indicate that several dropout subjects are improved at follow-up [30]. These data seem to highlight the difficulties of defining effective psychotherapy treatments [64-67].

## Conclusion

In conclusion, the fact that patients with EDs who do not accept or complete treatment are often more improved at follow-up than completers indicates that this should be a primary area for research on EDs.

Currently, though the results are still preliminary, a patient with a high risk profile for dropout is characterised by a diagnosis of binge-purging anorexia (inpatient setting) or a more severe bulimic symptomatology (outpatient setting) and tends to have borderline personality traits or a low ability to pursue his or her life's goals (low self-directedness).

These subjects with higher dropout risk seem to correspond to the subgroup of ED patients defined as "impulsive" [68] or "emotionally dysregulated" [69]. This subgroup of subjects is more frequent in individuals with diagnoses on the bulimia nervosa spectrum (AN-BP or BN diagnosis), who seem to have a genetic predisposition to greater psychiatric and personality comorbidity [70], poorer response to treatments [71] and a specific vulnerability to early life events [70]. This also seems to explicate the role of life events in predicting a higher risk of dropout in BN subjects: only a few researchers [72] have studied this aspect, but they found that parental breakup and childhood trauma (particularly sexual abuse) are significant predictors of early interruption of outpatient treatment.

In conclusion, the aims of this review were: (a) to create an incentive to adopt a shared definition of dropout in future studies, as proposed in the section entitled "Dropout definition", for inpatient and outpatient settings; (b) to motivate a wide-ranging assessment of the phenomenon of dropout, which should always include socio-demographic features, severity of the ED, nutritional status, dimensional assessment of personality (TCI or other similar instruments), Axis I and II comorbidity evaluation, eating and general psychopathology assessment with widely used psychometric scales, family assessment, life events and patients' perspective registration; (c) to create an incentive to use a kind of staging method for the assessment of readiness to change or similar issues in clinical practice; (d) the follow-up of dropouts; (e) the study of techniques and strategies to reduce the dropout phenomenon [23].

## Lists of abbreviations

DO: Dropout; DO-A: Dropout type A; DO-B: Dropout type B; E-DO: Early Dropout; L-DO: Late Dropout; FE: Failure to Engage; AN: Anorexia Nervosa; AN-R: Anorexia Nervosa Restrictor type; AN-BP: Anorexia Nervosa Binge Purging type; BN: Bulimia Nervosa; ED-NOS: Eating Disorders not Otherwise Specified; EDs: Eating Disorders; IBW: Ideal Body Weight; BMI: Body Mass Index; CBT: Cognitive Behavioural Therapy; PD: Personality Disorders; DSM-IV: Diagnostic and Statistic Manual of Mental Disorders IV; DSM-III-R: Diagnostic and Statistic Manual of Mental Disorders III Revised; For the tools abbreviations, see Additional file 3.

## Competing interests

The authors declare that they have no competing interests.

The authors declare a possible non-financial competing interest: "Two of the 26 papers reviewed were published by the study group that conducted this review".

## Authors' contributions

SF, ET, AP and GA-D, have made substantial contributions to conception, design and interpretation; ET has been involved in drafting and revising the manuscript; SF and GA-D gave final approval of the submitted version. AP and GA-D carried out the acquisition and analysis of data, whereas SF also tailored and made numerous revisions of the intellectual and scientific content of this paper.

All authors read and approved the final manuscript.

## Pre-publication history

The pre-publication history for this paper can be accessed here:



## Supplementary Material

Additional file 1 **main features of "dropout" studies included in the analysis: inpatient setting**. the data provided describe the features of inpatient studies included in this paper. Click here for file

Additional file 2 **main features of "dropout" studies included in the analysis: outpatient setting**. the data provided describe the features of outpatient studies included in this paper. Click here for file

Additional file 3 **assessment instruments and tools used in the studies included in this review**. a description of assessing instruments and tools is provided, with acronyms used in text and tables. Click here for file
