# Supplementary material for: Factors associated with dropout from treatment for eating disorders: a comprehensive literature review
Source: BMC Psychiatry. 2009 Oct 9;9:67. doi: 10.1186/1471-244X-9-67 (PMC2765944; doi:10.1186/1471-244X-9-67)
Supplement: Additional file 1 — main features of "dropout" studies included in the analysis: inpatient setting. the data provided describe the features of inpatient studies included in this paper. [file 1471-244X-9-67-S1.DOC]

| Year **Table 1. Main features of “dropout” studies included in the analysis: Inpatient setting** | **Authors** | Therapeutic **Setting**  **(Diagnosis)** | **Sample**  **(% dropout)** | **Age y**  **(mean ± SD)** | **Illness**  **Duration**  **y (mean± SD)** | **Treatments** | **Type of**  **Dropout***  **/Timing** | **Assessment** | **Predictors of dropout** |
| --- | --- | --- | --- | --- | --- | --- | --- | --- | --- |
| 1983 | Vandereycken W  and Pierloot R | Inpatients  (AN) | 133 (21.8% ED,  50% ED+LD) | 20.5  ± 4.8 | 3.1  ± 3.1 | D + CBT or  D + CBT + C, GT | DO-A  ED / LD | Clinical records  CGI | Later age of onset, older age,  lower socioeconomic status predicted the ED |
| 2001 | Kahn C  et al. | Inpatients  (AN-R, AN-BP) | 81 (33%) | 26.3  ± 7.4 | 7.8  ± 6.1 | D, CBT, MED, GT, C | DO-B | EAT-26, BSQ, BDI, SCL-90R, IIP-  C, RES | AN-BP and longer illness duration predicted dropout |
| 2004 | Woodside B  et al. | Inpatients  (AN-R; AN-BP) | 166 (51%) | 27.1  ± 9.0 | 6.7  ± 7.6 | D, PT, MED,FT | DO-B | Clinical records  EDE; EDI; BDI; PI; RES; FAM | AN-BP diagnosis, higher maturity fear, higher body mass index and depression, higher weight concern and lower restrictive attitudes, borderline traits |
| 2004 | Surgenor L  et al. | Inpatients  (AN) | 213 (20.2%) | 21.6  ± 7.1 | 5.2  ± 5.1 | n.m. | DO-A | Clinical records  BDI, EDI-2, EAT-26, RES | Lower body mass index, borderline traits , AN-BP diagnosis |
| 2005 | Zeeck A  et al. | Inpatients  (AN) | 133 (32%) | 24.8  ± 6.8 | 6.4  ± 5.9 | D, PP,  GT, CBT,  CO | DO-A  ED, MD,  LD | Clinical records  SCL-90R; EDI-2; IIP-C | Higher maturity fear, borderline traits, lower depression and number of previous hospitalisations predict dropout |
| 2006 | Carter JC et al. | Inpatients  (AN-R, AN-BP) | 77 (36%) | 25.5  ± 7.8 | 6.7±7.2 | D, GT | DO-A  DO-B | Clinical records | AN-BP with history of sexual abuse |
| 2007 | Masson PC et al. | Inpatients  (AN, BN, ED-NOS) | 186 (37.6%) | 26.5  ± 9.4 | 8.9  ± 7.5 | D, CBT, GT, FT, AT, RT | DO-A  (59%)  DO-B  (41%) | Chart reviews  RAI-MH, EDI-2, PAI | Psychiatric comorbidity (PTSD) |
| 2008 | Dalle Grave R. et al. | Inpatients  (AN, BN, ED-NOS) | 145(23.4%) | 25.6  ± 7.1 | 7.9  ± 6.4 | D, CBT. | DO-A | Clinical records  EDE, BDI, STAI, TCI | Persistence (TCI) |
| 2008 | Bewell CV and Carter JC | Inpatients  (AN-R; AN-BP) | 159 (40.2%) | 25.2  ± 7.5 | 5.9  ± 6.3 | D.GT | DO-A  (61%)  DO-B  (39%) | Clinical records  EDE, EDI. | Readiness to change |

**Note: n.m.=** datum not mentioned; **DO** = Dropout; **DO-A/DO-B =** Dropout of Kind A or B (see the text); **FE =** Failure to Engage; **ED** = Early Dropout; **MD** = Middle Dropout; **LD** = Late Dropout.

**PP =** Psychodynamic Psychotherapy; **D =** Diet/Nutritional Therapy; **CO** = Counselling to the Family; **FT** = Family Therapy; **CBT =** Cognitive Behavioural Therapy; **MED** = Medication; **PT** = Psychosocial Therapy; **GT =** Group Therapy; **IPT =** Interpersonal Psychotherapy; **ST** = Supportive Therapy; **AT** = Art Therapy; **RT** = Recreation Therapy.

**AN** = Anorexia Nervosa; **AN-BP** = Anorexia Nervosa Binge Purging subtype; **AN-R** = Anorexia Nervosa Restrictor subtype; **BN** = Bulimia Nervosa; **ED-NOS** = Eating Disorder Not Otherwise Specified (including Binge Eating Disorder).
