# Supplementary material for: Factors associated with dropout from treatment for eating disorders: a comprehensive literature review
Source: BMC Psychiatry. 2009 Oct 9;9:67. doi: 10.1186/1471-244X-9-67 (PMC2765944; doi:10.1186/1471-244X-9-67)
Supplement: Additional file 2 — main features of "dropout" studies included in the analysis: outpatient setting. the data provided describe the features of outpatient studies included in this paper. [file 1471-244X-9-67-S2.DOC]

| **Year**  **Table 2. Main features of “dropout” studies included in the analysis: Outpatient setting** | **Authors** | **Therapeutic**  **Setting**  **(Diagnosis)** | **Sample**  **(% dropout)** | **Age y**  **(mean ± SD)** | **Illness**  **Duration**  **y (mean± SD)** | **Treatments** | **Type of**  **Dropout***  **/Timing** | **Assessment** | **Predictors of dropout** |
| --- | --- | --- | --- | --- | --- | --- | --- | --- | --- |
| 1985 | Szmukler GI  et al. | Outpatients  (AN, BN) | 51 (27%) | 22.8  ± 8.0 | 3.9  ± 2.8 | PP  or FT | DO-A | MRAS, EAT, CCEI, CFI | Parental expressed emotion, BN diagnosis,  type of therapy-family therapy (multiple interactions) |
| 1992 | Van Strien DC  et al. | Outpatients  (AN, BN) | 90 (38%) | 17  ± n.m. | n.m. | n.a. | DO-A | Clinical records  MRAS; NPV | Lower educational level, higher hostility |
| 1993 | Coker S  et al. | Outpatients  (BN) | 31 (19%) | 28.0  ± 5.7 | n.m. | CBT | FE | Clinical records  EDE; BSQ; HDRS; SCQ; PDQ-R;  DEBQ | Presence of borderline personality disorder, lower self-esteem; bulimic symptoms |
| 1995 | Blouin J  et al. | Outpatients  (BN) | 81 (28.7%) | 25.9  ± 6.3 | 9.2 | CBT-GT | DO-A | BSCL, EDI, BDI, SCL-90R,  DSED,FES | Interpersonal distrust and difficulties relating to others |
| 1996 | Clinton N | Outpatients  (AN, BN, ED-  NOS) | 60 (36.6%) | 23.8  ± 5.8 | n.m. | FT  or PP or IPT or ST | DO-A | Clinical records  EDPET, EDI, CCEI | Differences between therapist and patient in  expectations about the treatment |
| 1996 | McKisack C &  Waller G | Outpatients  (BN) | 20 (26.6%;  25% FE) | 24.5  ± 2.2 | 5.8  ±1.9 | GT-CBT | DO-A | Clinical records  BITE, BSI, DES, SCANS, FAD-GF | Borderline and dissociative personality disorders  BITE severity score |
| 1997 | Waller G | Outpatients  (BN, AN-BP) | 50 (14% FE and  30% DO) | 21.8  ± 5.3 | n.m. | CBT | FE,  DO-A | Clinical records  EDI | Higher drive for thinness and body dissatisfaction, borderline traits predict dropout; completers had more severe levels of bulimic pathology |
| 2000 | Steel Z  et al. | Outpatients  (BN) | 32 (43%) | 23.0  ± 5.8 | 5.0  ± 4.0 | CBT | DO-A | Clinical records  EDI-2; BSQ; BDI; BHS; LCBS | Higher depression,  Higher external locus of control score |
| 2001 | Mahon J  et al. | Outpatients  (BN) | 111 (48%) | 24.4  ± 5.8 | n.m. | D + CBT or IPT | DO-A | Clinical records  SCL-90-R; EDI; RES | Lower age, previous psychiatric treatment,  employment status, parental breakup, childhood trauma |
| 2001 | Mahon J  et al. | Outpatients  (BN) | 114 (55.3%) | 26.7  ± 7.6 | n.m. | D + CBT or IPT | DO-A | Clinical records | Childhood trauma |
| 2001 | Wolk SL & Devlin  MJ | Outpatients  (BN) | 128 (34%) | n.m. | n.m. | CBT or IPT | DO-A | Clinical records  SCID-I, EDE, SOC | ---- |
| 2002 | Fassino S  et al. | Outpatients  (AN) | 99 (33%) | 21.9  ± 4.3 | 4.3  ± 3.8 | D, PP, CO | DO-A | Clinical records  EDI-2, TCI, STAXI | Higher anger expression and suppression, higher drive for thinness and social insecurity, higher harm avoidance, lower self-directedness and cooperativeness, borderline traits |
| 2003 | Fassino S  et al. | Outpatients  (BN) | 83 (30%) | 25.4  ± 5.7 | 6.8  ± 4.4 | D, PP,CO,  MED | DO-A | Clinical records  EDI-2, TCI, STAXI | Higher state anger and anger expression, higher impulsivity and maturity fear, lower self-directedness and cooperativeness (TCI), borderline traits |
| 2005 | Halmi K  et al. | Outpatients  (AN) | 122 (46%) | 24.8  ± 6.8 | n.m. | CBT  or CBT +  MED or MED | DO-A | Clinical records  Y-BOCS, SAS, MPQ, BDI, EDE, TFEQ, Y-BCEDS, RES, SEI | Lower self-esteem predicts dropout |
| 2005 | Swan-Kremeier  LA et al. | Outpatients  (AN, BN, ED-  NOS) | 139 (73%) | 27.0  ±11.1 | n.m. | D, CBT,  PT | DO-A | Clinical records | BN, ED-NOS, employment status (employed being more likely to drop out) |
| 2005 | Peake KJ et al. | Outpatients  Day Hospital  (EDs) | 261 (37.4%) | n.m. | n.m. | D, GT CBT, FT | DO-A | Clinical records  BDI, BAI, SCQ, SAS, EDE, EDI-2 | Impulsivity (EDI-2), body dissatisfaction, perfectionism, social adjustment scale, binge frequency, maturity fear. |
| 2007 | Morlino M et al. | Outpatients  (AN, BN, ED-  NOS) | 100 (53%) | 21.8  ± 5.6 | 3.9  ± 4.3 | D, PP, CO, MED | DO-A | Clinical records  EAT 40, EDI-2, CGI | Patient-therapist relationship |

**Note: n.m.=** datum not mentioned; **DO** = Dropout; **DO-A/DO-B =** Dropout of Kind A or B (see the text); **FE =** Failure to Engage; **ED** = Early Dropout; **MD** = Middle Dropout; **LD** = Late Dropout.

**PP =** Psychodynamic Psychotherapy; **D =** Diet/Nutritional Therapy; **CO** = Counselling to the Family; **FT** = Family therapy; **CBT =** Cognitive Behavioural Therapy; **MED** = Medication; **PT** = Psychosocial Therapy; **GT =** Group Therapy; **IPT =** Interpersonal Psychotherapy; **ST** = Supportive Therapy; **AT** = Art Therapy; **RT** = Recreation Therapy.

**AN** = Anorexia Nervosa; **AN-BP** = Anorexia Nervosa Binge Purging subtype; **AN-R** = Anorexia Nervosa Restrictor subtype; **BN** = Bulimia Nervosa; **ED-NOS** = Eating Disorder Not Otherwise Specified (including Binge Eating Disorder).
