# Supplementary material for: Factors associated with dropout from treatment for eating disorders: a comprehensive literature review
Source: BMC Psychiatry. 2009 Oct 9;9:67. doi: 10.1186/1471-244X-9-67 (PMC2765944; doi:10.1186/1471-244X-9-67)
Supplement: Additional file 3 — assessment instruments and tools used in the studies included in this review. a description of assessing instruments and tools is provided, with acronyms used in text and tables. [file 1471-244X-9-67-S3.DOC]

**Table 3. Assessment instruments and tools used in the studies included in this review**

(including acronyms used in the two previous tables)

| **Axis I and II diagnosis** | SCID-I (Structured Clinical Interview for Axis I)  SCID-II (Structured Clinical Interview for Axis II)  NIMH-DIS (National Institute of Mental Health-Diagnostic Interview) |
| --- | --- |
| **General psychopathology** | CGI (Clinical Global Impression)  SCL-90R (Symptom Checklist 90 Revised)  CCEI (Crown-Crisp Experiential Index)  RAI-MH (Resident Assessment Instrument-Mental Health)  BAI (Beck Anxiety Inventory) |
| **Depression** | HDRS (Hamilton Depression Rating Scale)  BDI (Beck Depression Inventory) |
| **Eating symptoms and attitudes** | EDI (Eating Disorder Inventory)  EDE (Eating Disorder Examination)  EAT-26 (Eating Attitudes Test)  BITE (Bulimic Investigatory Test)  SCANS (Setting Conditions for Anorexia Nervosa Scale)  MRAS (Morgan Russell Assessment Schedule)  DEBQ (Dutch Eating Behaviour Questionnaire)  BSCL (Bulimic Symptom Checklist)  DSED (Diagnostic Survey of Eating Disorders)  TFEQ (Three Factor Eating Questionnaire)  Y-BCEDS (Yale-Brown Cornell Eating Disorder Scale) |
| **Body Image and Body Shape** | BSQ (Body Shape Questionnaire) |
| **Self-Esteem and Self-Concept** | SCQ (Self Concept Questionnaire)  RES (Rosenberg Self-Esteem Questionnaire)  BHS (Beck Hopelessness Scale) |
| **Personality (functioning or dimensions)** | TCI (Temperament and Character Inventory)  BSI (Borderline Syndrome Index)  LCBS (Locus of Control of Behaviour Scale)  IIP-C (Inventory of Interpersonal Problems)  PDQ-R (Personality Diagnostic Questionnaire Revised)  MPQ (Multidimensional Personality Questionnaire)  NPV (Dutch Personality Questionnaire)  PAI (Personality Assessment Inventory) |
| **Patient’s point of view about treatment** | EDPET (Eating Disorder Patient’s Expectation of Treatment Questionnaire) |
| **Obsessivity** | PI (Padua Inventory)  Y-BOCS (Yale-Brown Obsessive Compulsive Scale) |
| **Anger** | STAXI (State Trait Anger Expression Inventory) |
| **Family dynamics** | CFI (Camberwell Family Interview)  FAM (Family Assessment Measure)  FAD-GF (Family Assessment Device)  Moos Family Environment Scale (FES) |
| **Other** | DES (Dissociative Experience Scale)  SAS (Social Adjustment Scale)  SEI (Side Effects Inventory)  SOC (Stage of Change Scale) |
